# Supplementary material for: Autoxidation Kinetics of Tetrahydrobiopterin—Giving Quinonoid Dihydrobiopterin the Consideration It Deserves
Source: Molecules. 2023 Jan 28;28(3):1267. doi: 10.3390/molecules28031267 (PMC9921404; doi:10.3390/molecules28031267)
Supplement: Supplementary file 1 [file molecules-28-01267-s001.zip › molecules-2120967-supplementary.pdf]

Supplementary Materials

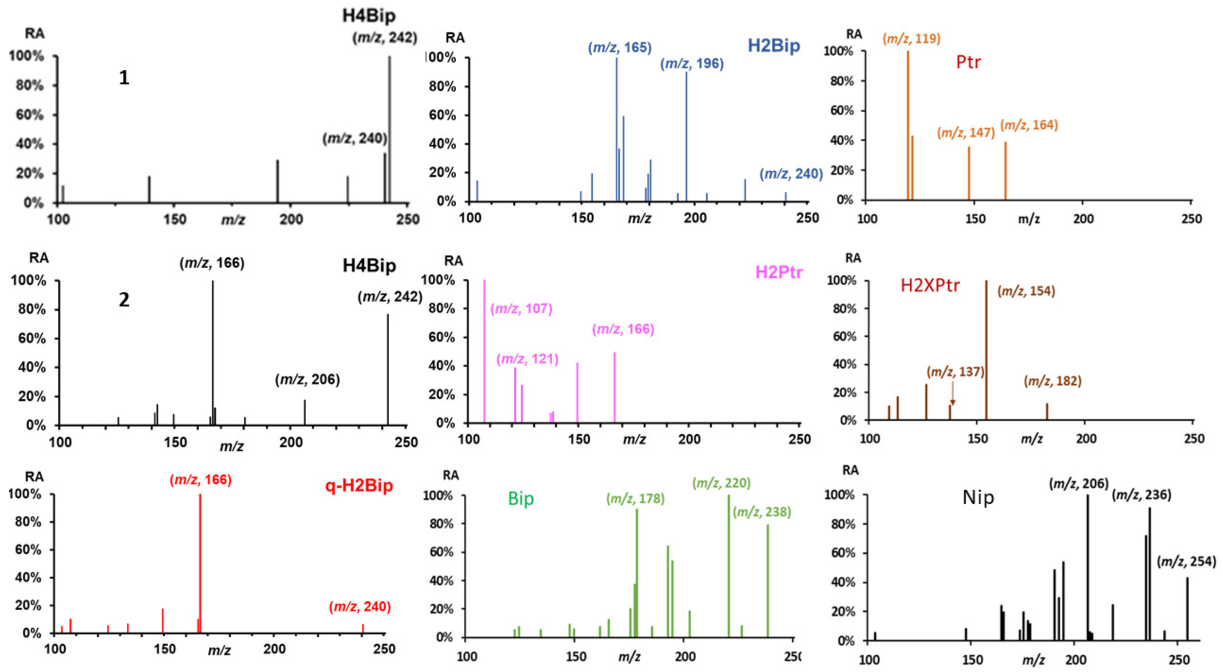

Figure S1. Extracted MS spectrum of H4Bip (1), and extracted MS/MS spectra of H4Bip (2), qH2Bip (Quinonoid dihydrobiopterin), H2Bip (7,8-Dihydrobiopetrin), H2Ptr (Dihydropterin), Bip (biopterin), Ptr (Pterin), H2XPtr (di-hydroxy-xanthopterin), and Nip (neopterin). Chromatographic conditions are detailed in the experimental section.

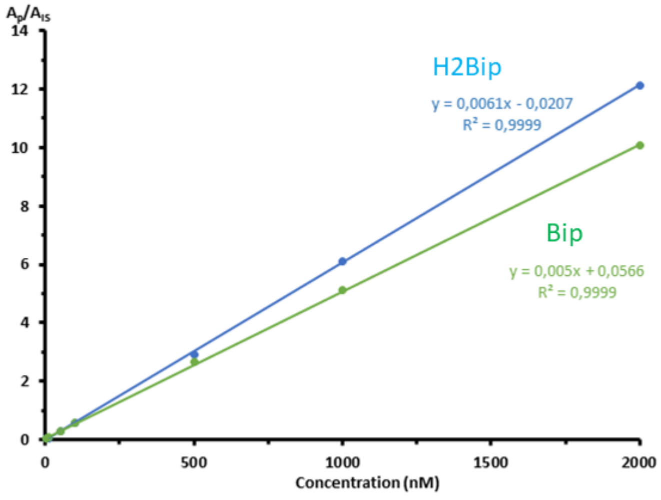

Figure S2. H2Bip and Bip quantification. Linearity of the method (5 -2000 nM, *n* = 6).
